# Prevalence, distribution and pattern of gastric lesions in slaughtered pigs in south-western Nigeria

**DOI:** 10.4102/ojvr.v83i1.1063

**Published:** 2016-05-26

**Authors:** Oladipo O. Omotosho, Benjamin O. Emikpe, Olalekan T. Lasisi, Theophilus A. Jarikre

**Affiliations:** 1 Department of Veterinary Medicine, University of Ibadan, Nigeria; 2 Department of Veterinary Pathology, University of Ibadan, Nigeria

## Abstract

Gastric lesions, especially ulceration, cause significant economic losses in the swine industry worldwide. The study was designed to assess its prevalence, distribution and pattern in pigs in south-western Nigeria. Slaughter house surveys were conducted on three government-established abattoirs in Lagos, Ogun and Oyo states. Stomachs from 480 pigs were assessed for gross lesions, which were graded using a modification of a standard technique. Tissues from different regions of the stomach were routinely stained to assess histopathologic changes. Data were presented as frequency counts and analysed using analysis of variance and chi-square technique. Significance was determined at *p* ≤ 0.05. Gastric lesions were encountered across the four regions of the stomach with a point prevalence of 57.29%. The prevalence of lesions in the non-glandular region was 32.9%, with severe hyperkeratosis (13.13%) being most frequently observed (*p* < 0.05). Erosions were significantly higher in the cardia (8.54%) (*p* < 0.05), followed by fundus (8.33%). Gastric ulcers were significantly higher in the fundus (19.58%) (*p* < 0.05). Scars of healed ulcers and lacerations were also observed in the fundus (5.42%) (*p* < 0.05). The gastric lesion distribution across the four regions of the stomach and the occurrence of ulceration in the fundus showed an unusual pattern, which is rarely reported in other parts of the world. The reason for these findings in pigs in Nigeria is not fully understood; therefore, further studies are required to identify and manage these factors for increased productivity, improved animal welfare and enhanced food security.

## Introduction

Gastric lesions in pigs, especially ulceration, are an ongoing problem resulting in high morbidity and mortality in many herds and significant economic losses in the swine industry worldwide (Friendship 2003). Gastric ulceration in pigs is usually diagnosed at post-mortem, although a few studies in veterinary practice have reported its diagnosis by endoscopic examination (Ayles *et al*. 1999; Mackin *et al*. 1997). Slaughter house surveys over many years in different regions of the world have revealed high prevalence of gastric lesions in the pars oesophagea which may be as high as 100% (Christensen & Cullinane 1990; Elbers, Vos & Dirkzwager 1995a; Straw *et al*. 1992). It may also occur in the glandular region (cardia, fundus and antrum) (Monteiro 2011). Gastric lesions often encountered include hyperkeratosis, erosions, ulcerations and gastric neoplasms. Most of the available studies report the occurrence of ulcers in the non-glandular region in pigs (Kopinski & McKenzie 2007; Ramis *et al*. 2004), with only a few reports on its occurrence in the glandular region (Monteiro 2011). The frequency of ulcers in the pars oesophagea is believed to have increased with the introduction of confinement rearing and the use of grain-based processed rations in the diet of pigs (Friendship 1999).

The major economic concern associated with gastric ulceration is sudden death from bleeding gastric ulcers, the most common cause of mortality during the grower-finisher stage (Friendship 2003; Melnichouk 2002). Less acute blood loss may result in anaemic and unthrifty pigs, whilst the process of healing may produce scars leading to occlusion of the oesophageal opening causing difficulty in feed passage into the stomach (Friendship 2003). Gastric ulceration is also known to have a depressive effect on daily live weight gain in finishing pigs (Elbers *et al*. 1995b). However, the economic implication of this condition in the Nigerian swine population is unknown.

The prevalence and pattern of gastric lesions in the swine population in Nigeria is scanty in literature, except for a focal study reported by Majekodunmi *et al*. (2013). The present study was therefore designed to assess the prevalence, distribution and pattern of gastric lesions in pigs in south-western Nigeria.

## Materials and methods

### Study area

Slaughter house surveys were conducted in three government-owned abattoirs in south-western Nigeria between the months of July and September 2013. The abattoirs are located at Oko-oba, Agege, Lagos State (Lat. 6.664608°, Long. 3.691406°), Oke-aro in Ogun State (Lat. 6.689691°, Long. 3.332572°) and Ibadan, Oyo State (Lat. 7.420805°, Long. 3.923755°). These slaughter houses are the main government-designated abattoirs for pork processing in these three states, which are major pig-producing areas in south-western Nigeria.

### Sampled pigs

The pigs sampled were those presented to the abattoir from individual small- and medium-scale farms within the state and from neighbouring communities. The breeds of pig were Large white, Landrace, Duroc and cross-breeds with weight ranging between 30 kg and 110 kg. The ‘market ready pigs’ presented to the abattoirs were subjected to ante-mortem inspection and only apparently healthy pigs were passed for slaughter.

### Sampling technique

A simple random sampling of a total of 480 stomachs was conducted during a 6-week period (2 weeks per location) of regular abattoir activities. The stomachs were examined grossly for lesions across the four (pars oesophagea, cardia, fundus and antrum) regions of the pig stomach. The sample size required was determined using the International Fund for Agricultural Development sample size calculation tool:

$$n=\frac{t^{2}\times p(1-p)}{m^{2}},$$ [Eqn 1]

where *n* = required sample size; *t* = confidence level at 95% (standard value of 1.96); *p* = estimated prevalence in the project area, which was estimated at 46% (0.46) because the prevalence in a previous preliminary study in Nigeria was 46% (Majekodunmi *et al*. 2013); *m* = margin of error at 5% (standard value of 0.05); *n* = (1.96)^2^(0.46)(0.54)/0.05^2^, *n* = 381.54.

The adequate sample size is 381.5. In the study, 480 stomachs were sampled.

### Sample preparation and lesion assessment

Freshly collected stomachs were cut open along the lesser curvature and inverted. The mucosal surface was gently cleaned with running water to ensure only the ingesta were removed. The presenting gross changes were assessed quantitatively. The lesion type, distribution and pattern were noted and scored based on severity using a six-point ordinal scoring system (0–5), which is a modification of the visual morphological scoring guide for oesophagogastric ulcers (Kopinski & Mckenzie 2007).

The scores are 0 indicating no lesion, 1 indicating slight keratosis, 2 indicating severe keratosis and thickened epithelium, 3 indicating erosions, 4 indicating mucosa damage (lacerations and scars) and 5 indicating ulceration. The oesophagogastric lesion scoring guide was modified to accommodate lesions of the glandular stomach as the initial scoring guide was designed strictly for lesions in the pars oesophagea.

Tissue samples were collected randomly from the different regions of the stomach from lesions and healthy tissues. They were preserved in 10% buffered formalin and processed routinely.

Haematoxylin and eosin, Giemsa and Warthin–Starry silver stains were used to assess morphology and the presence of organisms.

### Statistical analysis

The lesion occurrence was presented as frequency counts and percentages. Lesion scores were analysed using analysis of variance and chi-square. Significance was determined at *p* ≤ 0.05.

## Results

**FIGURE 1:**
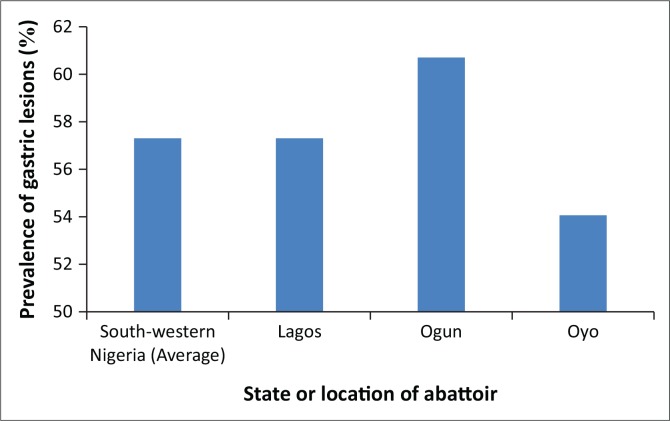
Prevalence of gastric lesions in pigs in south-western Nigeria.

The gastric lesions encountered in these slaughter house surveys included hyperkeratosis, erosions, damage to gastric mucosa (lacerations and scars) and active ulcers, which varied in size, shape and distribution across the stomach regions. The overall prevalence of gastric lesions was 57.29% and 32.90% in the whole stomach (i.e. all regions combined) and non-glandular region (pars oesophagea), respectively. Although occurrence of gastric lesions was observed in stomachs surveyed in the three slaughter houses (Lagos: 57.22%, Ogun: 60.67% and Oyo: 54%) (Figure 1), no gross neoplastic lesion was encountered and there was no carcass or organ condemnation because of these lesions.

**FIGURE 2:**
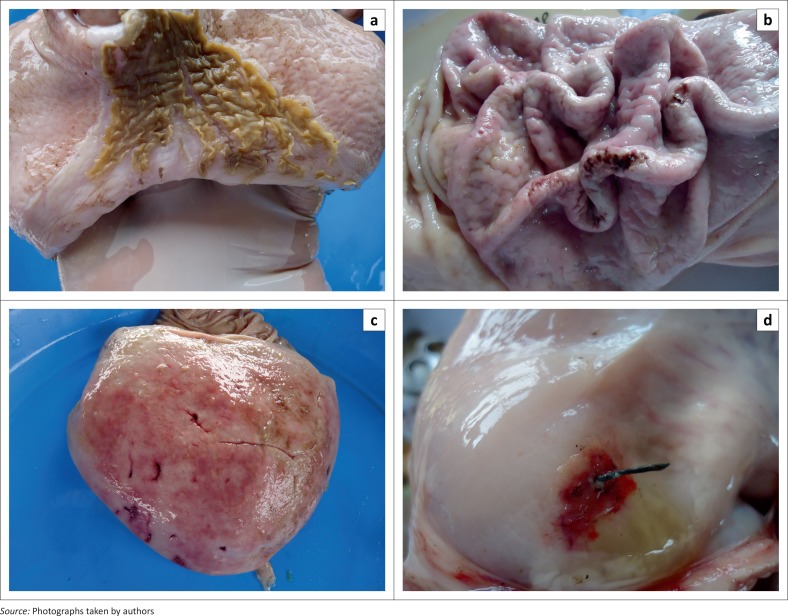
(a) Hyperkeratosis of the pars oesophagea with areas of erosion and ulceration. (b) Multiple ulcers of the fundus. (c) Multiple lacerations of the fundic gland region. (d) Mucosal and muscular layer damage by sharp metal at the antral region projecting into the peritoneal cavity.

Table 1 shows the occurrence and distribution of lesions across the four regions of the pig stomach. The pars oesophagea had lesions in 32.90% of the tissues examined, with severe hyperkeratosis (13.13%) being significantly higher than slight hyperkeratosis (9.38%), erosion (7.08%), mucosal damage (0.83%) and ulcers (2.5%). Instead of the smooth, white, glistening mucosal surface expected of the non-glandular epithelium of the pars oesophagea, often observed are roughened appearance (parakeratosis) which was often yellow in colour as a result of bile staining in a similar pattern to what has been previously described (Friendship, 2003). In some instances, erosions and ulcers were detected between the ridges when parakeratosis is present (Figure 2a).

**TABLE 1:** Occurrence and distribution of lesions in the different regions of the pig stomach in south-western Nigeria.

Region of the stomach	Lesion	Lagos		Ogun		Oyo		SW Nig. (Total)		*p*-value
			
		*n*	%	*n*	%	*n*	%	*n*	%	
Pars oesophagea	No gross lesion	114	63.33	101	67.33	107	71.33	322	67.08	0.03*
	Slight hyperkeratosis	19	10.56	14	9.33	12	8.00	45	9.38	-
	Severe hyperkeratosis	21	11.67	21	14.00	21	14.00	63	13.13	-
	Erosion	19	10.56	8	5.33	7	4.67	34	7.08	-
	Mucosal damage	1	0.56	0	0.00	3	2.00	4	0.83	-
	Ulcers	6	3.33		6	4.00	0	0.00	12	2.50	-
Cardia	No lesion	149	82.78	114	76.00	135	90.00	398	82.92	0.00*
	Erosion	18	10.00	15	10.00	8	5.33	41	8.54	-
	Mucosal damage	6	3.33	4	2.67	5	3.33	15	3.13	-
	Ulcers	7	3.89	17	11.33	2	1.33	26	5.42	-
Fundus	No lesion	126	70.00	93	62.00	100	66.67	319	66.46	0.04*
	Erosion	15	8.33	17	11.33	8	5.33	40	8.33	-
	Mucosal damage	10	5.56	3	2.00	13	8.67	26	5.42	-
	Ulcers	29	16.11	37	24.67	28	18.67	94	19.58	-
Antrum	No lesion	168	93.00	134	89.33	138	92.00	440	91.67	0.17
	Erosion	2	1.11	4	2.67	4	2.67	10	2.08	-
	Mucosal damage	6	3.33	2	1.33	5	3.33	13	2.71	-
	Ulcers	4	2.22	10	6.67	3	2.00	17	3.54	-

*SW Nig., South-western Nigeria.

* Data in bold font are statistically significant at *p* ≤ 0.05

In the cardia, erosion of the glandular mucosa was the most frequently encountered lesion (8.54%). Ulcers (5.42%) and mucosal damage (3.13%) were also observed. Lesions were observed in 33.54% of the fundus with the occurrence of ulcers (19.58%) being significantly higher than erosions (8.33%) and mucosal damage (5.42%). The antrum was least affected with lesions (8.33%) having erosions (2.08%), mucosal damage (2.71%) and ulcers (3.54%). Hyperkeratosis was entirely restricted to the pars oesophagea. Analysis of lesion scores in each stomach region in individual states and across the south-west Nigeria showed that the fundus had a significantly higher lesion score than other stomach regions (Table 2).

**TABLE 2:** Lesion severity across the different regions of the pig stomach as observed in the three states of south-western Nigeria.

Stomach region state	Pars oesophagea	Cardia	Fundus	Antrum
Oyo	0.57 ± 1.02^b^	0.36 ± 1.11^b^	1.44 ± 2.12^b^	0.31 ± 1.09^b^
Ogun	0.74 ± 1.27^b^	0.97 ± 1.79^a^	1.65 ±2.20^b^	0.47 ±1.38^b^
Lagos	0.83 ± 1.32^b^	0.63 ± 1.42^b^	1.28 ± 2.01^b^	0.28 ± 1.06^b^
*SW Nig. (Total)	0.72 ± 1.22^b^	0.65 ± 1.48^b^	1.45 ± 2.11^a^	0.35 ± 1.18^c^

Note: Superscript a > b along the column for each stomach region in individual states and along the row for South-western Nigeria.

SW Nig., South-western Nigeria.

*p* ≤ 0.05

**FIGURE 3:**
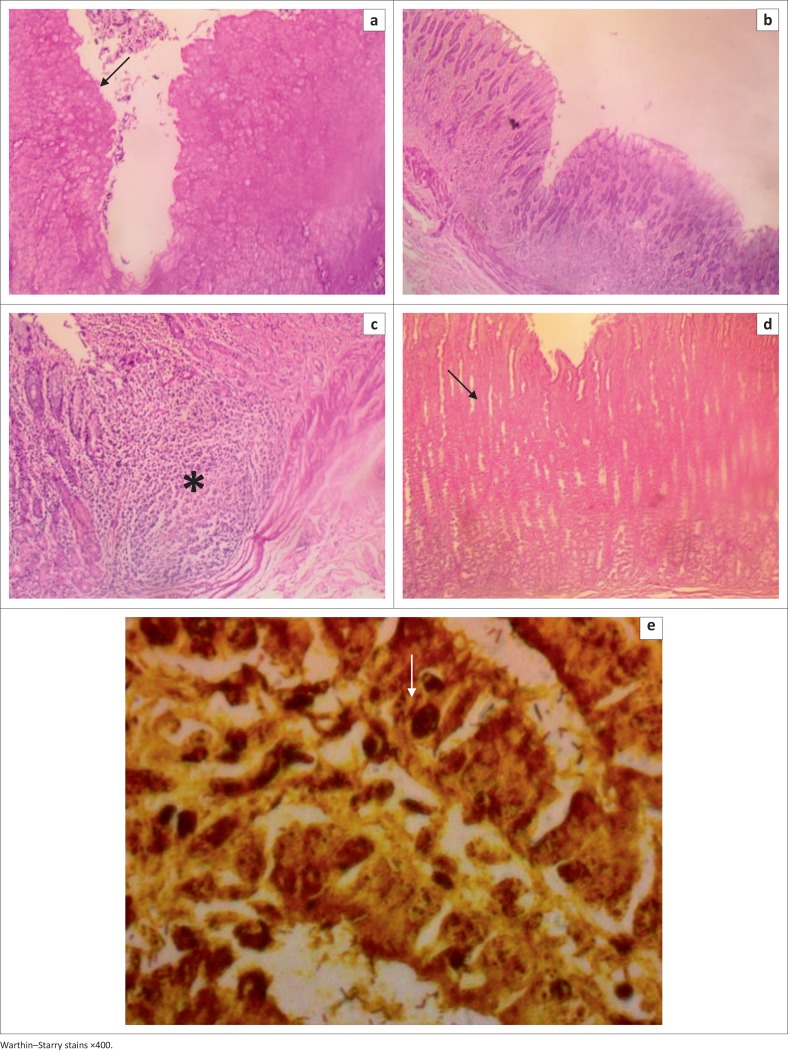
Pars oesophagea: (a) showing mucosal defects (ulcers) and parakeratosis (arrow); Cardia: (b), (c) showing diffuse gastritis and gut-associated lymphoid tissue hyperplasia (asterisk); Fundus: (d) showing mucosal defects and necrosis of neck cells (arrow); (e) showing comma-shaped bacteria (arrow).

The histological evaluation of the gastric lesions showed ulcerations (Figure 3e) and diffuse gastritis (Figure 3f) with accompanying gut-associated lymphoid tissue hyperplasia (Figure 3g). The fundus also revealed mucosal defects and necrosis of neck cells (plate2k) with the presence of bacteria rods demonstrated by Giemsa and Warthin–Starry stains (Figure 3l).

## Discussion

The study reports the prevalence, unusual occurrence and distribution of lesions across the four regions of the pig stomach at three abattoirs in South-western Nigeria. The prevalence of the gastric lesions in the study (57.29% for all stomach regions combined and 32.90% in the pars oesophagea) and its widespread occurrence presents that one to two of every three slaughter-age pig is likely to have a form of gastric lesion. This high and widespread occurrence shows its significant burden in the swine industry in south-western Nigeria. Gastric lesions are known to lead to tangible production losses through slow growth and unthriftiness. These losses are usually because of reduced feed intake and blood loss, which when acute leads to mortality. The finding is similar to earlier reports of a range of 30% – 80% prevalence of gastric lesions related to ulcerous processes in slaughtered pigs with on-farm mortalities often above 1% (Gonzalo 2010; Swaby & Gregory 2012). No gross neoplastic lesion was encountered during these surveys. This may be because of ante-mortem inspection at the abattoirs, which ensures that only apparently healthy animals were slaughtered.

The stomach of pigs is known to be fragile especially at the pars oesophagea, which is characterised by stratified squamous epithelium than in the glandular region (cardia, fundus and antrum). The epithelial type coupled with the lack of protective secretions make this region more prone to gastric ulcers than the glandular region (Doster 2000). The pars oesophagea may present with hyperkeratosis, which if the process continues, leads to erosion, ulceration, blood loss and death (Gonzalo 2010). In the study, hyperkeratosis of different grades – slight (9.38%) or severe (13.13%) – was the major lesion observed in the pars oesophagea and it was restricted to this region of the stomach. Hyperkeratosis is known to be a pre-ulcerous lesion that occurs because of abnormalities of keratinisation caused by conditions disturbing the intricate control over the gastric epithelium (O’Sullivan *et al*. 1996; Roels & Ducatelle 1997). Gross erosion mostly affected the cardia and the fundus, whilst lacerations and scars were more frequently observed in the fundus. The finding of scars is evidence of previous active ulcerous or traumatic lesions, as gastric ulceration is known to be a dynamic process that may occur rapidly and possibly heal almost as fast (Friendship 2003).

It is worth noting that the frequent occurrence of foreign bodies in the stomach contents including sharp objects may be a contributing factor to the occasional finding of lacerations. In a particular instance, a metal object was observed to have pierced through the muscular layer of the stomach to the peritoneum. These foreign objects in addition to causing gastric lesions may also lead to obstruction, peritonitis or septicaemia. This further highlights the importance of feed hygiene and nutrient balance in swine husbandry as this occurrence may be because of unhygienic feed preparation or mineral deficiency causing high foreign object content of feeds or pica, respectively. There is need for production-stage studies for better understanding of other factors that may be responsible for the frequent occurrence of foreign bodies in the pig stomach in this environment.

Apart from the unusual occurrence of ulcerative and traumatic lesions in the fundus, the analysis of the cumulative lesion scores also shows that the most severe lesions affected the fundic gland region. This finding is in contrast to that of other researchers who have reported that gastric lesions in pigs are often restricted to the pars oesophagea (Friendship 1999; Melnichouk 2002). Ulceration of the pars oesophagea, also termed oesophagogastric ulceration, has several predisposing factors including feeding practices, fineness of feed particles, feed nutrient content, infections and stress (Amory, Mackenzie & Pearce 2006; Wondra *et al*. 1995). Few older studies have reported the presence of these lesions in the glandular stomach, which some associated with certain systemic diseases such as salmonellosis, erysipelas or hog cholera infection (Monteiro 2011). The histopathology further elucidates the gross findings and revealed the possible involvement of bacteria in the pathogenesis of the ulceration in pigs. A further study of the role of sub-clinical diseases, pica and other factors peculiar to this region may provide information that will be beneficial in the prevention and management of gastric lesions in pigs.

Although there was no offal trim or organ condemnation at the abattoirs because of gastric lesions as they do not cause any known health risk to humans, it remains a production disease whose effect on swine productivity significantly reduces profit from the swine industry and poses a risk to the food security of the growing population in Nigeria.

## Conclusion

Gastric lesions are a significant problem in swine husbandry in south-western Nigeria having an unusual pattern of distribution in the stomachs. There is need for further research into peculiar causative and contributory factors responsible for this high burden and pattern of occurrence. Early diagnosis especially through non-invasive techniques will support better management of the condition with a view to improving animal welfare in swine production and enhancing food security in Nigeria.
